# Glances at the Hospitals

**Published:** 1900-01-06

**Authors:** 


### GLANCES AT THE HOSPITALS.

### WEST SUSSEX INFIRMARY AT CHICHESTER.

THE last annual report of this hospital shows that it has continued its work of usefulness during the year ending September, 1899. The highest number resident at any one time was 57, and the total number admitted since the opening of the hospital was 21,570. The average number in daily residence was 40, and the average number of days each patient was in the house was 35. The total number treated during the year was 378, and of these 152 were medical cases admitted by ticket, 124 were surgical cases similarly admitted, and 102 were accidents. Of the total number under treatment, namely, 415, we learn that 223 were discharged cured, 98 relieved, 17 neither better nor worse, 29 died, 1 discharged at his own request, 2 were sent to the Isolation Hospital, and 45 remained on the books. A new lodge for the porters has been built, and it was found necessary to incur other expenses connected with the household, but these have been met by the sums falling in as legacies. The private nursing branch has been satisfactorily managed by the matron, Miss Speck, and has proved to be a source of income to the hospital. The governors, under the chairmanship of Mr. Legge, are to be congratulated on the results of the year, financial and otherwise, and especially so when we point out that the reserve account has £18,500 in Consols, and that the convalescent fund amounts to £165.

